# Comparing the harmful effects of nontuberculous mycobacteria and Gram negative bacteria on lung function in patients with cystic fibrosis^☆^

**DOI:** 10.1016/j.jcf.2015.09.007

**Published:** 2016-05

**Authors:** Tavs Qvist, David Taylor-Robinson, Elisabeth Waldmann, Hanne Vebert Olesen, Christine Rønne Hansen, Inger Hee Mathiesen, Niels Høiby, Terese L. Katzenstein, Rosalind L. Smyth, Peter J. Diggle, Tania Pressler

**Affiliations:** a Copenhagen Cystic Fibrosis Center, Department of Infectious Diseases, Rigshospitalet, Copenhagen University, Denmark; b Department of Public Health and Policy, University of Liverpool, Liverpool, UK; c Department of Medical Informatics, Biometry and Epidemiology, Friedrich-Alexander-Universität Erlangen-Nürnberg, Germany; d Aarhus Cystic Fibrosis Center, Department of Pediatrics, University Hospital Skejby, Aarhus, Denmark; e Copenhagen Cystic Fibrosis Center, Department of Pediatrics, Rigshospitalet, Copenhagen University, Denmark; f Copenhagen Cystic Fibrosis Center, Department of Clinical Microbiology, Rigshospitalet, Copenhagen University, Denmark; g Institute of Child Health, University College London, London, UK; h Faculty of Medicine, Lancaster University, Lancaster, UK

**Keywords:** ATS, American Thoracic Society, CF, cystic fibrosis, CFRD, cystic fibrosis related diabetes, CI, confidence interval, %FEV1, forced expiratory volume in 1 s expressed as % of predicted, IDSA, Infectious Disease Society of America, MABSC, *Mycobacterium abscessus* complex, MAC, *Mycobacterium avium* complex, NTM, nontuberculous mycobacteria., Lung function, Abscessus, NTM, Gram negative, CF

## Abstract

**Background:**

To better understand the relative effects of infection with nontuberculous mycobacteria and Gram negative bacteria on lung function decline in cystic fibrosis, we assessed the impact of each infection in a Danish setting.

**Methods:**

Longitudinal registry study of 432 patients with cystic fibrosis contributing 53,771 lung function measures between 1974 and 2014. We used a mixed effects model with longitudinally structured correlation, while adjusting for clinically important covariates.

**Results:**

Infections with a significant impact on rate of decline in %FEV1 were *Mycobacterium abscessus* complex with − 2.22% points per year (95% CI − 3.21 to − 1.23), *Burkholderia cepacia* complex − 1.95% (95% CI − 2.51 to − 1.39), *Achromobacterxylosoxidans* − 1.55% (95% CI − 2.21 to − 0.90), and *Pseudomonas aeruginosa* − 0.95% (95% CI − 1.24 to − 0.66). Clearing *M*. *abscessus* complex was associated with a change to a slower decline, similar in magnitude to the pre-infection slope.

**Conclusions:**

In a national population we have demonstrated the impact on lung function of each chronic CF pathogen. *M. abscessus* complex was associated with the worst impact on lung function. Eradication of *M. abscessus* complex may significantly improve lung function.

## Graphical abstract

**Figure:**
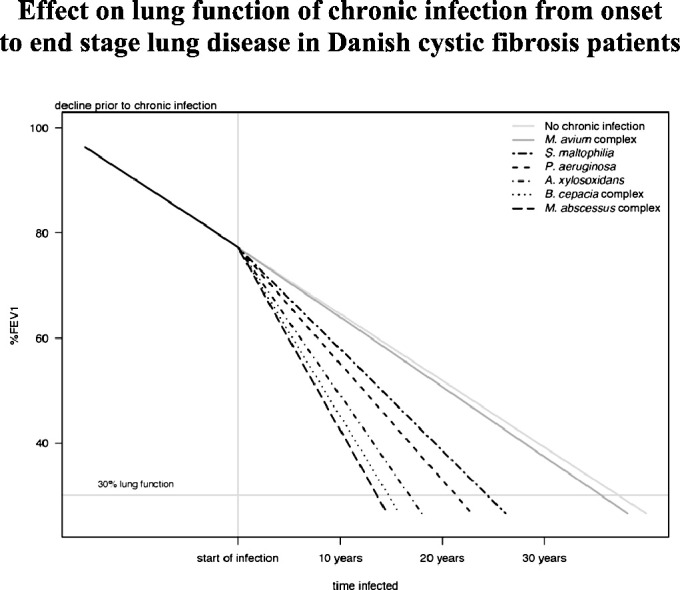

## 1 Introduction

The natural history of CF lung disease is characterized by chronic progression with intermittent episodes of acute worsening of symptoms, termed pulmonary exacerbations, often precipitated by bacterial infections, which become established within viscid airway secretions. Understanding the distinct impact of chronic infections in cystic fibrosis (CF) is important, because lung function decline takes place in a setting of multiple competing pathogens. Prioritizing treatment starting with the most serious threat to patients' health is a central challenge for clinicians. While the impact of major Gram negative infections have previously been reported [1], [2], there are limited data from population level studies comparing the relative influence of the major bacterial pathogens.

The principle of always using early, aggressive treatment aimed at eradication of both Gram positive and negative infection has been in use since 1976 in Denmark [3], [4]. As a consequence chronic persistent *Staphyloccocus aureus* infection is infrequently seen [5]. Methicillin-resistant *S. aureus* is rare in Denmark in general and almost non-existent among patients with CF [5].

If eradication therapy for Gram negative bacteria fails, elective 2-week courses of intravenous chemotherapy are administered at regular intervals to pre-empt exacerbations and maintain lung function. Since 1987 inhaled antibiotics have been a part of the standard treatment of chronic infection with subsequent additions of continuous dornase alpha and azithromycin treatment, when these treatments became available. These principles have been employed for infection due to *Pseudomonas aeruginosa*, *Achromobacter xylosoxidans* and *Burkholderia cepacia* complex, which have all been described to be associated with worse outcomes, including survival, lung function, and nutritional status [1], [6], [7], [8], [9]. The significance of chronic infection with *Stenotrophomonas maltophilia* and nontuberculous mycobacteria (NTM) is less clear. Except in rare cases, *S. maltophilia* is not treated in Denmark [10], while NTM is subject to intensive treatment following guidelines from the American Thoracic Society (ATS) and the Infectious Disease Society of America (IDSA)'s consensus documents [11]. Three previous studies of clinical outcomes following NTM have shown, respectively, no effect, significant effect or a calamitous effect on expected forced expiratory volume in 1 s (%FEV1) [12], [13], [14]. Understanding the significance of NTM on lung function in patients with cystic fibrosis (CF) is important, given the intensive therapeutic regimen required to clear infection, and associated side effects [11], [15].

The aim of the study was to assess and contrast the impacts of chronic Gram negative infections and NTM on lung function in patients with CF. *Aspergillus* infection and Gram positive bacterial infection were not included in this dataset due to inherent differences in how chronic infection was defined and registered for these pathogens.

## 2 Methods

We undertook a longitudinal analysis of lung function in Danish patients with CF. All patients born from 1974 onwards were included if seen at the Copenhagen CF Center; patients seen at the other Danish CF center in Aarhus, were included from 2002. The pre-1974 birth cohorts were excluded to reduce the influence of survivor bias as previously described in this dataset [8]. Post-transplantation data were likewise excluded. Further methodological details are available in the online appendix.

### 2.1 Setting

Throughout the study period from 1974 to August 2014, patients attending the two Danish CF Centers were seen routinely every month in the outpatient clinic, for evaluation of clinical status, pulmonary function, and microbiology of lower respiratory tract secretions. Pulmonary function tests were performed according to international recommendations [16], measuring FEV1, expressed as a percentage of predicted values for sex and height using reference equations from Wang or Hankinson [17], [18].

The hypothesis we wanted to test was that onset of infection with *Mycobacterium abscessus* complex (MABSC) and *Mycobacterium avium* complex (MAC) would lead to deterioration of lung function as previously shown with *P. aeruginosa*, *A. xylosoxidans*, *B. cepacia* complex and *S. maltophilia*. The primary outcome of interest in the statistical model was the change in slope of %FEV1 at the first time point, at which, individuals transitioned to fulfill the definition of chronic infection. Chronic Gram negative infection was defined according to modified Leeds criteria (more than 50% positive culture samples during a year). In Copenhagen, specific precipitating antibodies were also used to support the definition of chronic infection as previously described [4], [19], [20]. For NTM, the term chronic was not used, but we distinguished between patients who fulfilled the ATS/IDSA's criteria for NTM pulmonary disease [11], and those who did not. Onset of NTM infection was defined as the date of first recorded positive NTM culture. Clearing infection was defined as consistent culture negativity in > 4 NTM cultures over a minimum of 12 months following cessation of NTM treatment. Throughout the period, patients were screened routinely for NTM during bronchoalveolar lavages and when clinically indicated. Between 1987 and 1988 and again from 2011 onward, all patients with CF in Copenhagen were also screened annually for NTM with mycobacterial culture.

### 2.2 Statistical analysis

We developed a longitudinal model for the data using a previously published approach [8], [9]. In brief, we developed a multivariate longitudinal model to assess the association between onset (for all infections) and offset of infection (in the case of MABSC), and slope of lung function trajectory, while adjusting for birth cohort, genotype (coded as the number of delta F508 alleles (0, 1 or 2)); pancreatic insufficiency (PI) (coded 0 or 1 as a baseline covariate); and CF related diabetes (CFRD) diagnosed using previously published criteria (coded 0 or 1 as a time-varying covariate) [21]. The final model assumed a linear function for the population-averaged time-trend, though we explored non-linear approaches, which did not improve model fit (Appendix). The longitudinally structured correlation was modeled as an exponentially decaying function of time difference (Appendix) [8]. This approach provides a more realistic estimate of the %FEV1-trend of patients with chronic lung disease by taking into account the imprecision and correlation in repeated measurements on the same individual over time [21]. We assessed the influence of each infection individually first, and then in a mutually adjusted model containing all of the infections. We further assessed the effect of co-infection by adding interaction terms between the infections to the model. We estimated model parameters by maximum likelihood, using generalized likelihood ratio statistics to compare nested models when building the final multivariate model and the Akaike information criterion (AIC) to compare non-nested models (when testing for the significance of infection interaction terms); and Wald statistics to test hypotheses about model parameters [22]. We visualized the model parameters of interest by plotting population averaged %FEV1 trajectories with other model parameters held constant. As a robustness test we repeated the analysis dropping the earliest birth cohort due to the potential for survivor effects in the earlier birth cohorts. A level of 0.05 was set for statistical significance. R version 3.1.1 was used for the analysis (http://www.R-project.org).

### 2.3 Ethical considerations

The study was approved by the Danish Data Protection Agency (file no. 2008-41-2682).

## 3 Results

### 3.1 Population characteristics

The dataset contained 53,771 lung function measures on 432 patients who visited the Danish CF centers between 1974 and 2014. The median number of %FEV1 measures per person was 100 (range 1–530). The median follow-up period was 12.3 years (range 0–35.5), with a total of 9250 person-years of follow-up (see Appendix for further details). Seventy-six patients were followed for more than 30 years. The baseline characteristics of the population, stratified by birth cohort are shown in Table 1. Genotype and CFRD were not significant in the final multivariable model, and were therefore dropped (appendix).

### 3.2 Chronic infections

*P. aeruginosa* was the most common infection, followed by *S. maltophilia*, *A. xylosoxidans* and *B. cepacia* complex. Eighty-six (20%) patients developed two chronic infections (Table 2). Forty-four patients were culture positive for MABSC at least once; 14 patients had MAC and two had both MABSC and MAC infection. NTM patients contributed a total of 251 NTM positive person years to the study with a mean NTM positive follow-up time of 7.6 years. Thirty-nine (70%) patients cleared their NTM infection.

### 3.3 Effect of infections on lung function

**Fig. 1:**
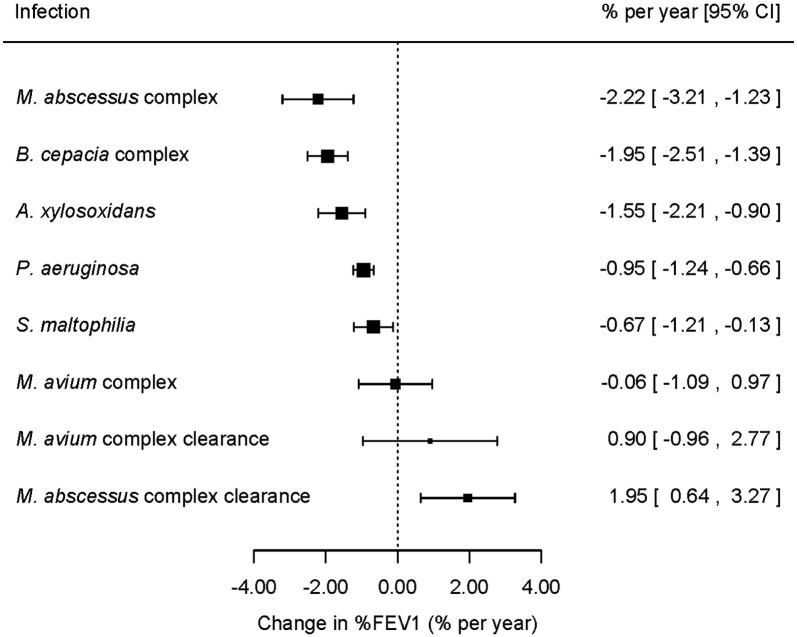
Change in the rate of decline of lung function (%FEV1) following onset of each infection and clearance of nontuberculous mycobacterial infection. The point sizes are drawn proportional to the precision of the estimates.

With the exception of MAC, onset of all infections was associated with a significant acceleration in %FEV1 decline. On the basis of the point estimates, after adjustment for demographic, genetic and clinical factors, MABSC had the largest effect on lung function; − 2.22 percentage points per annum (95% CI − 3.21 to − 1.23), followed by the *B. cepacia* complex − 1.95 (95% CI − 2.51 to − 1.39), *A. xylosoxidans* − 1.55 (95% CI − 2.21 to − 0.90), *P. aeruginosa* − 0.95 (95% CI − 1.24 to − 0.66), and *S. maltophilia* − 0.67 (95% CI − 1.21 to − 0.13) (Fig. 1). An overall Wald test confirmed that there were general significant differences between the effect sizes of the six infections (p < 0.001). The effect of multiple infections was checked by adding interactions between the infections to the model in cases where there were more than 10 people with co-infection (see Table 2). None of the interaction terms were significant or improved the model AIC.

**Fig. 2:**
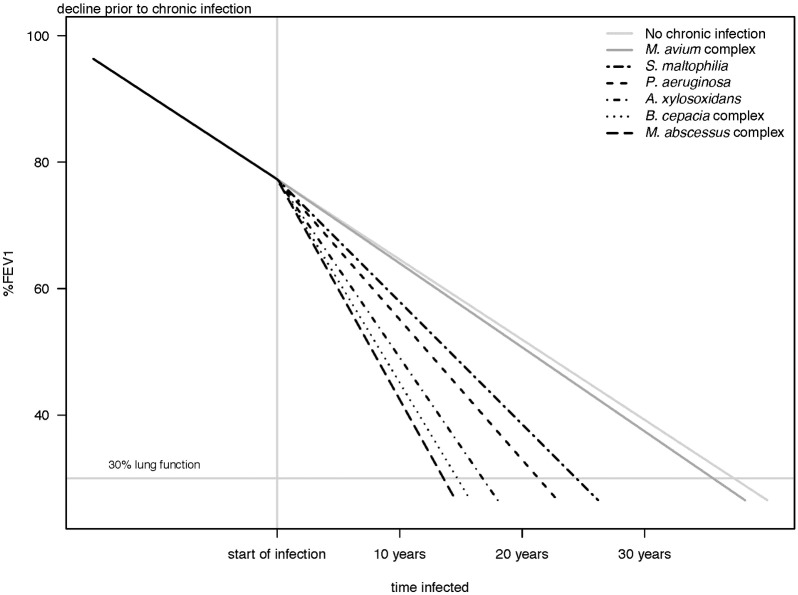
Effect on lung function of chronic infection from onset to end stage lung disease in Danish cystic fibrosis patients. The figure visualizes the impact of onset of chronic infections by plotting population averaged %FEV1 trajectories before and after onset of infection with other model parameters held constant. Thus for a patient born in 1994 and infected at age 20, all other things being equal, end stage lung disease will occur after 13.6, 14.7, 16.8, 21.3, 24.4 and 35.6 years for MABSC, *B. cepacia* complex, *A. xylosoxidans*, *P. aeruginosa*, *S. maltophilia* and MAC respectively. Footnote: Trajectories plotted and held constant for a person born in the 1994–2004 birth cohort, mutually adjusted for other co-variates in the model, with onset of infection occurring at age 20.

Projecting the trend of the negative change in lung function for each CF pathogen into estimated time to the development of end stage lung disease, Fig. 2 illustrates the clinical consequence of each chronic infection. Thus, for a patient born in 1994 and infected at age 20, all other things being equal, end stage lung disease will occur after 13.6, 14.7, 16.8, 21.3, 24.4 and 35.6 years for MABSC, *B. cepacia* complex, *A. xylosoxidans*, *P. aeruginosa*, *S. maltophilia* and MAC respectively.

### 3.4 MABSC and lung function decline

**Fig. 3:**
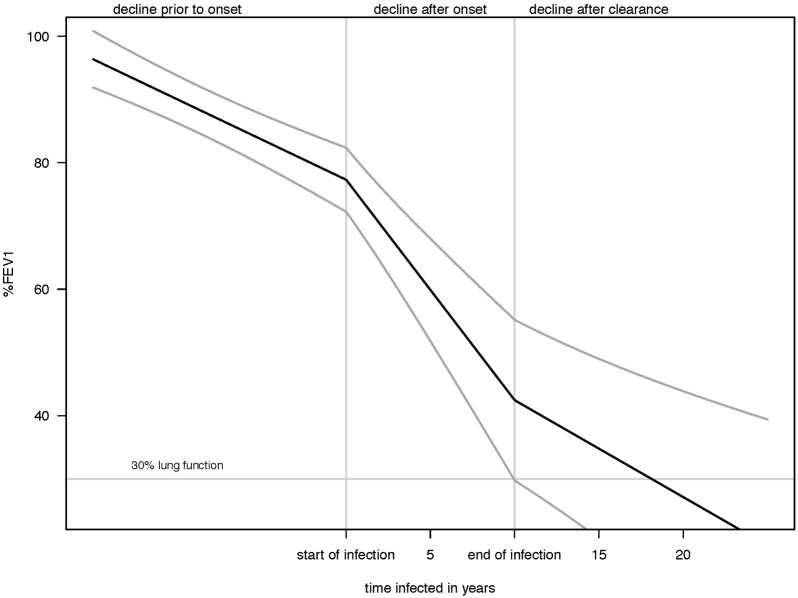
Mean effect on lung function of *Mycobacterium abscessus* complex infection from onset and clearance of the infection in Danish patients with CF. Footnote: Trajectories plotted and held constant for a person born in the 1994–2004 birth cohorts, mutually adjusted for other co-variates in the model, and extended beyond the range of the observed data to demonstrate progression to end stage lung disease.

Onset of MABSC was associated with the steepest decline in lung function decline, but clearance of the infection was associated with a change to a slower decline, similar in magnitude to the pre-infection decline (Fig. 1, Fig. 3).

### 3.5 Robustness tests

Repeating the analysis only on data from patients born after 1984 and patients who fulfilled the ATS/IDSA criteria did not markedly change the results. Tests of the model fit are reported in the appendix.

## 4 Discussion

This is the first population level study to simultaneously compare the effects of different infections on the longitudinal rate of decline of lung function in CF. The strength of this analysis is the visit frequency of examinations (every month) and long period of follow-up in a complete national population, facilitating precise estimation of the time of onset of infection. Furthermore, the frequency of measures of the %FEV1 dataset allowed a more sophisticated statistical model to be used, which leads to more robust estimation of the rate of lung function decline [8]. The order of the negative effects on lung function was (worst to best): MABSC, *B. cepacia* complex, *A. xylosoxidans*, *P. aeruginosa*, and *S. maltophilia.*

### 4.1 NTM and lung function decline

With 21 years of follow-up of patients with NTM, this study provides strong evidence for the effects of MABSC and MAC on lung function in CF. MABSC demonstrated an effect of − 2.2% of excess lung function decline per year; in contrast MAC infection had no significant effect. Previous studies have confirmed that MABSC is more virulent than MAC in patients with CF [11], [23], [24], but only three studies have looked at effects on lung function decline in CF. In 2003, Olivier et al. [12] showed that 18 patients with CF and ATS/IDSA defined NTM disease did not have a significantly accelerated lung function decline over a 15-month period. They suggested that a longer observation time might reveal a difference. In 2010, Esther et al. [13] examined longitudinal data from 23 patients with MABSC and found an excess lung function decline of − 0.78% per year. Martiniano et al. [14] followed 70 MAC and 24 MABSC cases for three years after NTM infection and found excess annual rates of decline of − 4.1% for those who fulfilled ATS/IDSA criteria, and − 1.6% per year for those with only one positive culture. The authors did not distinguish between the effects of MABSC and MAC. In our analysis we did differentiate between the two species, but interestingly could not find an isolated effect of fulfilling ATS/IDSA criteria for NTM pulmonary disease. This could be due to an underestimation of patients who fulfilled the ATS/IDSA microbiological criteria, either from the early part of the cohort, where patients were cultured less frequently or due to the increase in MABSC cases seen in the last year of the study, where some new cases with clinical deterioration, did not have sufficient time to fulfill the criteria. Scandinavian NTM outcomes, including clearance rates have been published recently [25], with approximately half of ATS/IDSA defined NTM infected patients achieving culture negativity, typically, but not exclusively, following extensive treatment. Patients with MABSC were more likely to develop end stage lung disease and the clinical significance of a first positive NTM culture was high, with almost 3 out of 4 eventually progressing from a first positive culture to a second [25].

### 4.2 Effect of clearing MABSC

The observed significant slower decline in lung function following MABSC clearance (1.9% improvement per year) was similar to the effect size of acquisition (− 2.2% per year). To our knowledge, this is the first study to suggest that excessive loss of lung function may be mitigated by MABSC clearance and our observation of two entirely opposite effects after onset and clearance of disease give further weight to the notion of a causal association between MABSC infection and lung function decline [26].

### 4.3 Gram negative infection

The observed influence of the different Gram negative infections on lung function decline is not surprisingly in accordance with previous independent analyses from the Danish CF population [10], [27], [28], [29]. Apart from *A. xylosoxidans*, the results are also in accordance with reports from other countries, which ascribes *B. cepacia* complex the worst role, albeit with some inter-species variation [30], followed by *P. aeruginosa*, for which there is a larger body of evidence [2], [31] and finally *S. maltophilia*, which is not considered to be a serious cause of lung function decline [32], [33], although this perception has been challenged [34]. *A. xylosoxidans* has been reported to have either no impact on lung function [35], or an effect indistinguishable from that of *P. aeruginosa* [36]. We did not demonstrate any interaction between infections in terms of their effects on lung functions, but this finding may reflect insufficient numbers to detect such effects. The omission of chronic Gram positive bacteria and *Aspergillus* infection was based on respectively very low prevalence and difficulties in comparing criteria for chronicity. The issue of whether the appearance of NTM in CF is the cause of, or alternatively the consequence of, deteriorating lung function is a key question. The reverse effect in lung function we observed following clearance of MABSC is compelling evidence that it is indeed the mycobacteria causing the deterioration and not the other way around. Limitations in the study are the retrospective design and the risk of ascertainment bias which could underestimate the mitigating effect of asymptomatic MABSC on the overall MABSC effect. Confounding independent of the variables we adjust for can likewise not be ruled out. Differences in how NTM infection and Gram negative infection are defined and screened for, can be expected to be built into any CF study since disease criteria and screening recommendations are dissimilar. Our robustness testing however suggested these differences were not important, as the same effect of MABSC was seen in children infected late in the period and independently of fulfilling ATS/IDSA criteria. The influence of survivor bias on lung function estimates in the earlier birth cohorts is a common problem in datasets of this type [37]. We handled this by excluding patients born before 1974 [8], where treatment regimens changed frequently. Repeating the analysis using only data from more recent cohorts did not alter the results. Furthermore, fitting the mixed effects longitudinal model by maximum likelihood implicitly takes this drop out into account and generates the parameter estimates that one would expect to see if dropout had not occurred [38], [39].

### 4.4 Conclusions

In summary, we have demonstrated the marked effect on lung function of each Gram negative CF pathogen and NTM, allowing for the first time, to compare the infections in degrees of severity. We have shown how MABSC has an important negative effect on lung function, whereas we did not detect a significant effect for MAC infection. We observed that clearing MABSC may restore lung function decline to its previous slope, pointing to the important role of eradication therapy in cystic fibrosis.

## Conflict of interest

DTR, EW and PD received funding from the Medical Research Council.

## Contributions

Guarantors: DTR, TQ;

Conception and design: DTR, TQ, TP, RLS;

Data collection: HVO, IHM, CRH, TLK, NH;

Analysis and interpretation: DTR, EW, PJD, TQ; and

Drafting the manuscript for important intellectual content: TQ, DTR, EW, HVO, CRH, IHM, NH, TLK, RLS, PJD, TP.

## Figures and Tables

**Table 1:** Characteristics of Danish cystic fibrosis patients by birth cohort.

	1974 n (%)	1984 n (%)	1994 n (%)	2004 n (%)	Total n (%)
Cohort	120 (27.8)	133 (30.8)	123 (28.5)	56 (13)	432 (100)
Female	62 (51.7)	68 (51.1)	62 (50.4)	31 (55.4)	223 (51.6)
Pancreatic insufficiency	112 (93.3)	123 (92.5)	120 (97.6)	52 (92.9)	407 (94.2)
*P. aeruginosa*	82 (68.3)	34 (25.6)	22 (17.9)	3 (5.4)	141 (32.6)
*S. maltophilia*	14 (11.7)	22 (16.5)	21 (17.1)	5 (8.9)	62 (14.4)
MABSC	10 (8.3)	22 (16.5)	9 (7.3)	3 (5.4)	44 (10.2)
MAC	5 (4.2)	5 (3.8)	4 (3.3)	0 (0)	14 (3.2)
*A. xylosoxidans*	7 (5.8)	31 (23.3)	1 (0.8)	1 (1.8)	40 (9.3)
*B. cepacia* complex	22 (18.3)	13 (9.8)	2 (1.6)	0 (0)	37 (8.6)

**Table 2:** Number of cystic fibrosis patients in Denmark co-infected with Gram negative bacteria or nontuberculous mycobacteria.

	*P. aeruginosa*	*B. cepacia* complex	*S. maltophilia*	*A. xylosoxidans*	MABSC(cleared)	MAC(cleared)
*P. aeruginosa*	141	13	18	8	17 (10)	4 (2)
*B. cepacia*	–	37	2	1	0	0
*S. maltophilia*	–	–	62	6	15 (5)	4 (2)
*A. xylosoxidans*	–	–	–	40	6 (0)	3 (3)
MABSC	–	–	–	–	44 (20)	2
MAC						14 (8)

MABSC = *Mycobacterium abscessus* complex, MAC = *Mycobacterium avium* complex.
