# Supplementary material for: Comparing the harmful effects of nontuberculous mycobacteria and Gram negative bacteria on lung function in patients with cystic fibrosis
Source: J Cyst Fibros. 2016 May;15(3):380–5. doi: 10.1016/j.jcf.2015.09.007 (PMC4893021; doi:10.1016/j.jcf.2015.09.007)
Supplement: Supplementary file 1 — Supplementary material. [file mmc1.docx]

**Online data supplement**

**Comparing the harmful effects of nontuberculous mycobacteria and Gram negative bacteria on lung function in patients with cystic fibrosis**

Tavs Qvist^§^, David Taylor-Robinson^§^, Elisabeth Waldmann, Hanne Vebert Olesen, Christine Rønne Hansen, Inger Hee Mathiesen, Niels Høiby, Terese L Katzenstein, Rosalind L Smyth, Peter Diggle, Tania Pressler

^§^ Contributed equally

This data supplement contains additional information on the statistical methods employed in the study. In addition, further plots and results are presented.

**Methods**

*Subjects*

Cystic fibrosis (CF) was diagnosed as either two known CF-causing mutations in the CF transmembrane conductance regulator (CFTR) gene, and/or two positive sweat-tests together with symptoms compatible with the disease. Lung function testing was generally measured pre-bronchodilator treatment, although, consistent with normal practice, patients were not instructed to omit their bronchodilator on the day of the measurement.

*Nontuberculous mycobacteria (NTM)*

Only small changes in mycobacterial culture methodology occurred during the period, notably the addition of the MGIT™ (Mycobacteria Growth Indicator Tube) 960 system and species-level identification by 16-23S spacer array technique and sequencing of the rpoB gene.

*Statistical analysis*

*Model*

We use a linear mixed effects model with longitudinally structured correlation. This allows a flexible specification of the mean response and incorporates three qualitatively different components of stochastic variation about the mean response ([1](#_ENREF_1), [2](#_ENREF_2)).

Let *Y_ij_* denote the *j*th repeated measurement (here, %FEV1) on the *i*th patient, and write

*Y_ij_ =μ_ij_ +R_ij_,* (1)

where *μ_ij_* is the mean, population-averaged, response and *R_ij_* is the stochastic variation about the mean response.

In (1), the mean response is specified as a linear combination of explanatory variables, hence

$\mu_{ij}=\alpha+\sum_{k=1}^{p} x_{ijk}\beta_{k}$ (2)

In (2), the *x_ijk_* can be any measured values, whether time-constant or time-varying; for example, sex or age. Despite the model’s title, non-linear effects can also be captured. Polynominal time-trends can be defined by including powers of age amongst the *x_ijk_*. Spline functions can be obtained by including both powers of age and indicator variables at selected time-points, called knots. For example, a model in which *x_ij1_ =age* and *x_ij2_ = 0* for age less than *10*, taken to be the age of onset of infection, *x_ij2_ = age − 10* for age greater than *10*, defines a linear spline with a single knot, also called a split-line or broken-stick model, with a change in slope at age of onset of infection at age 10 years.

To complete the model-specification we decompose the stochastic term *R_ij_* in (1) into three components, hence

*R_ij_ = U_i_ + W_i_(t_ij_) + Z_ij_,* (3)

where *t_ij_* is the *j*th measurement time for the *i*th patient and the three components of *R_ij_* are specified as follows. Firstly, *U_i_* describes how the average lung function of the *i*th patient varies about the population-averaged response for all patients with the same values of the explanatory variables *x_ijk_*, for example all males aged 20 years. The model assumes that the *U_i_* are independent copies of a Normally distributed random variable with mean zero and variance *ν^2^*. Secondly, the stochastic process *W_i_(t)* describes how the actual lung function of the *i*th patient varies over time. The model assumes that the *W_i_(t)* are independent copies of a stationary Gaussian process with mean zero, variance *σ^2^* and correlation function *ρ(u) = Corr{W_i_(t), W_i_(t−u)}* ([2](#_ENREF_2)). Typically, *ρ(u)* decays towards zero as *u* increases. In the current application, we use an exponential correlation function, *ρ(u) = exp(−|u|/φ)*, in which the parameter *φ* describes the rate at which the correlation decays towards zero with increasing time-separation, *u*. The exponential correlation function is a special case of the Matérn family, which includes a second parameter that allows the correlation function *ρ(u)* to assume different shapes if the exponential model does not give a good fit ([3](#_ENREF_3)). Thirdly, *Z_ij_* describes how the imperfectly measured lung function of the *i*th patient at their *j*th measurement time, *t_ij_*, differs from their underlying actual lung function, i.e. measurement error. In principle, the properties of the measurement error could be estimated directly by repeated measurement of %FEV1 within a single follow-up session. In practice, the *Z_ij_* represent the sum of two sources of variation: pure measurement error and within-patient variation in lung-function on shorter time-scales than the shortest time-interval between successive measurement times, *t_ij_* and *t_i,j+1_*. The model assumes that the *Z_ij_* are independent copies of a Normally distributed random variable with mean zero and variance *τ^2^*.

*Exploratory analysis*

Exploratory analysis consists of identifying a suitable form for the set of mean responses *μ_ij_* and obtaining initial estimates of the parameters in the model for the stochastic terms *R_ij_*.

For the first of these tasks, we use a combination of ordinary least squares fitting of a regression model, and kernel smoothing. Ordinary least squares gives unbiased estimates of baseline explanatory variable effects whatever the structure of the *R_ij_*, whilst kernel smoothing allows the investigation of possibly non-linear time-trends after adjustment for baseline effects. A kernel smoother is an estimate of the form

$$s(t)=\sum_{i} \sum_{j} r_{ij}w_{ij}$$

in which the *r_ij_* are the residuals from the regression on baseline explanatory variables whilst the smoothing weights *w_ij_* are scaled to add to 1 and are proportional to *f(t − t_ij_)*, where the kernel function, *f(u)*, is a probability density function symmetric about *u* = 0; a common choice is a Normal probability density function with mean zero and standard deviation *h*. In exploratory analysis, the value of *h* can be chosen subjectively so as to obtain a smoothly varying estimate *s(t)*.

For the second task, we first re-define the residuals *r_ij_* to adjust for the estimated smooth time-trend *s(t)* as well as for baseline explanatory variables. To estimate the covariance structure of these residuals we use the variogram, whose definition is as follows. Let *v_ijk_ = (r_ij_ − r_ik_)^2^/2* and *u_ijk_ = |t_ij_ − t_ik_|*. Pick a grouping interval *h*, let *n_r_* be the number of *u_ijk_* that lie between *(r − 1)h* and *rh* and $\bar{v}_{r}$ the sample mean of the corresponding *v_ijk_*. A plot of $\bar{v}_{r}$ against *(r − 0.5)h* is called the *sample variogram (*[*1*](#_ENREF_1)*)*. It estimates the function *V(u) = τ^2^ + σ^2^{1 − ρ(u)}*, called the theoretical variogram. The sample variance of the residuals estimates the quantity *τ^2^ + ν^2^ + σ^2^*. Hence, as illustrated in Figure 1 below, by sketching a smooth curve to fit the sample variogram we can obtain initial estimates of the variance components *τ^2^, ν^2^* and *σ^2^*, and of the correlation function *ρ(u)*.

*Confirmatory analysis*

We estimate all of the model parameters by maximum likelihood estimation. This requires numerical maximisation of the log-likelihood function, whose algebraic form is that of the logarithm of a multivariate Normal probability density function with mean vector specified by (2) and a block- diagonal covariance matrix in which the *i*th block has diagonal elements *c_jj_ = τ^2^ + ν^2^ + σ^2^* and off-diagonal elements *c_jk_ = ν^2^ + σ^2^ρ(u_ijk_)*.

To compare nested models (i.e. one is a special case of the other), we use generalized likelihood ratio tests. If *L_1_* and *L_0_* denote the maximised values of the log-likelihood for nested models with *p* and *p − q* parameters, the generalised likelihood ratio test for the goodness-of-fit of the simpler model compares *D = 2(L_1_ − L_0_)* with critical values of the chi-squared distribution on *q* degrees of freedom.

To test hypotheses about model parameters, we use Wald tests. These exploit the property that the maximum likelihood estimates are approximately unbiased and Normally distributed, with standard errors that can be computed from the fitted model; for the algebraic details, see Diggle, Heagerty, Liang and Zeger ([1](#_ENREF_1)).

The fitted theoretical variogram *V(u)* gives a graphical representation of the estimated variance components *τ^2^, ν^2^ and σ^2^*, and of the correlation function *ρ(u)*.

*Goodness-of-fit*

To test the overall goodness-of-fit of the final model, we analyse the residuals as follows. Firstly, plots of residuals against fitted values should show random scatter. Secondly, the residuals should have approximately the same covariance structure as the fitted model, which we check by comparing their sample variogram with the theoretical variogram of the model.

**Results**

*Visualizing the dataset*

The spaghetti plots in Figure E1 illustrates randomly selected individual %FEV_1_ traces from the analysis dataset, illustrating the high follow-up frequency over long periods of time.

Figure E1: Five randomly selected %FEV_1_ profiles from the Danish dataset

Figure E2 shows the frequency and length of follow up for CF patients in the dataset.

**Figure E2: Histogram of frequency and duration of follow up**

***Exploring the form for the population average***

Figure E3 shows all the %FEV1 measures in the Danish dataset over time in a scatterplot, with an added mean smoother in red. The mean smoother does not take into account the correlation of repeated measures within individuals. There appears to be a fairly linear decline in mean %FEV1 to about age 25, where the mean stabilizes, before becoming more erratic at older ages where the sample size is smaller. A piecewise ordinary least squares (OLS) regression (blue line) with a change in slope at age 25 provides an improved fit over a straight line, and by eye one can see that this fits the smoother well. However, when one fits the same piecewise mean in the longitudinal model using MLE, then the change in slope at age 25 is not significant (green line). This indicates that the levelling off of the mean smoother to some extent reflects selective drop out (death). The final longitudinal model implicitly takes this drop out into account and generates the parameter estimates that one would expect to see if dropout had not occurred. We therefore modelled the population average as a straight line.

***Fig E3: All data with smoothed mean trend***

***
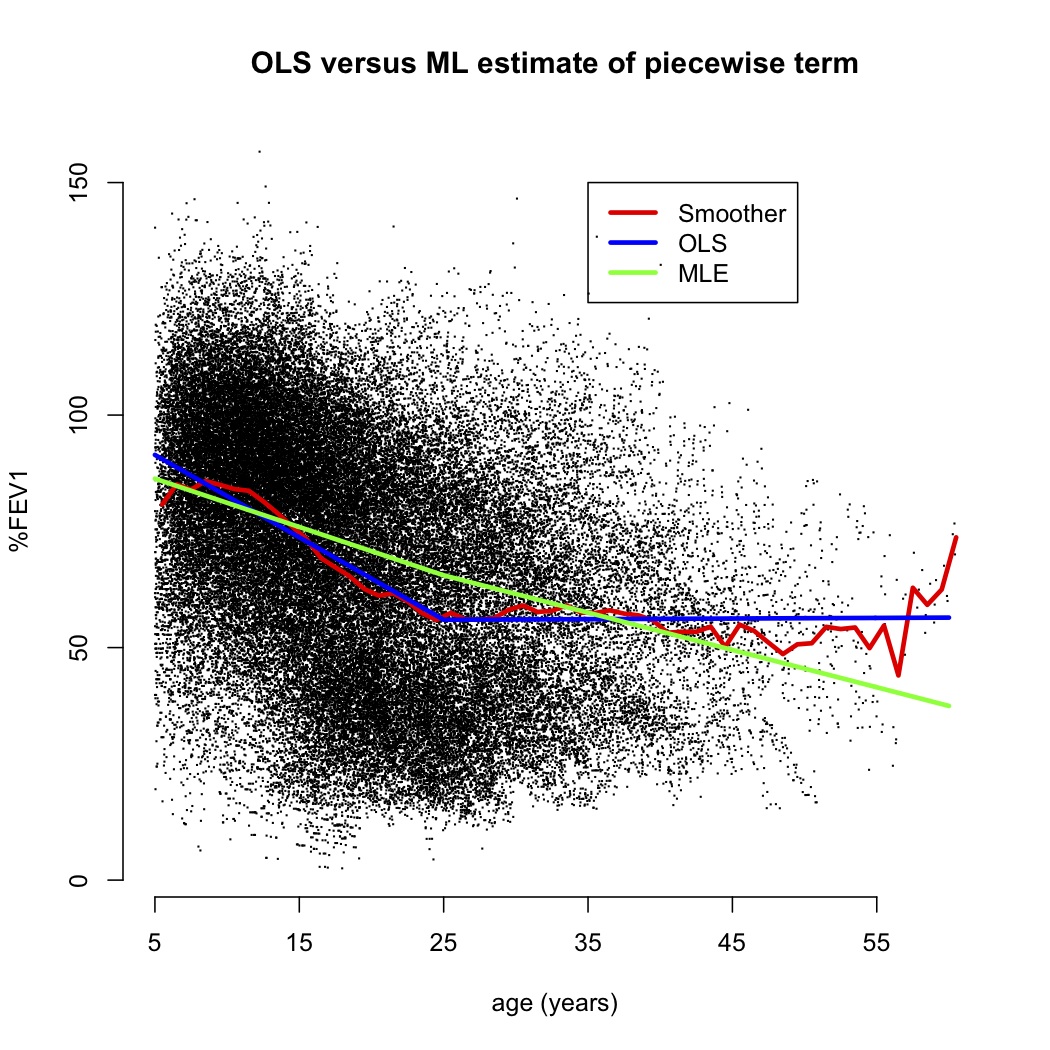
***

McKay et al propose a novel approach to modelling lung function decline in an adult population([4](#_ENREF_4)), involving a linear mixed effect model with a cubic spline to account for non-linear population-averaged decline in lung function with age and a standard random intercept and slope model to account for within-patient variability. The spline gives a more flexible set of models for the population-averaged trajectory, but our analysis of the Danish dataset shows that the random intercept and slope model is too rigid to capture the pattern of within-patient variability in %FEV1 over longer periods. Our approach can easily be combined with the spline model to describe non-linear population-averaged decline, since the form of the population average and the within-patient correlation structure are separate issues. Indeed, building on our modelling approach Szczesniak et al combine a non-parametric approach to the population average, with serial correlation, in the context of a mixed model applied to US registry data ([5](#_ENREF_5)). However, as illustrated above in figure E3 this was not needed for the Danish dataset. Note also that an adequate model for the correlation structure in the data is necessary for prediction at the individual patient level.

*Assessing Model fit*

Figure below compares the empirical variogram fit to the theoretical variogram plotted using the MLE estimates from the lme() function in R, with an exponential correlation. This shows that the modelled correlation function approximates reasonably to the empirical correlation in the dataset.

**Figure E3 : Comparison between empirical variogram and the maximum likelihood variogram estimate**

The estimated variance (%FEV1 ^2^) components derived from the modelled variogram are as follows: total variance=485 within-person variance=122, between-person variance=317, error variance=46.

*Residual diagnostics*

Figure E4 plots the standardized residuals against the fitted values. There are no trends in the residuals, and there is no evidence of non-constant variance.

**Figure E4 : Scatterplot of standardized residuals versus fitted values**

Figure E6 illustrates the same as Figure 1 in the main article, but with each covariate added singly instead of together.

**Figure E5: Ranked effect sizes showing the change in the rate of decline in %FEV1 associated with onset and clearance of chronic – from models with infections added singly**

**Table E1: Parameter estimates with standard errors and p-values for full model**

|  | Parameter estimate | Standard error | p-value |
| --- | --- | --- | --- |
| Intercept | 90.4527 | 6.0706 | 0.0000 |
| Age | -0.2789 | 0.3268 | 0.3934 |
| Sex | 2.3211 | 2.1968 | 0.2913 |
| cohort1974 | -12.4104 | 3.0142 | 0.0000 |
| cohort1994 | 10.0760 | 2.9189 | 0.0006 |
| cohort2004 | 4.9758 | 3.6642 | 0.1752 |
| Pancreatic insufficiency | -4.1647 | 5.9568 | 0.4848 |
| *P. aeruginosa* | -0.9501 | 0.1455 | 0.0000 |
| *S. maltophilia* | -0.6696 | 0.2773 | 0.0157 |
| *B. cepacia* | -1.9500 | 0.2846 | 0.0000 |
| *A. xylosoxidans* | -1.5529 | 0.3356 | 0.0000 |
| MABSC | -2.2168 | 0.5046 | 0.0000 |
| Cleared MABSC | 1.9543 | 0.6695 | 0.0035 |
| MAC | -0.0607 | 0.5238 | 0.9078 |
| Cleared MAC | 0.9022 | 0.9515 | 0.3430 |
| Age*sex | 0.0294 | 0.1321 | 0.8241 |
| Age*cohort1974 | 0.5762 | 0.1716 | 0.0008 |
| Age*cohort1994 | -0.5763 | 0.2319 | 0.0129 |
| Age*cohort2004 | 1.0595 | 0.6866 | 0.1228 |
| Age*pancreatic insufficiency | -0.4147 | 0.3215 | 0.1971 |

**Table E2: Parameter estimates with standard errors and p-values for model with only data for patients born after 1984**

|  | Parameter estimate | Standard error | p-value |
| --- | --- | --- | --- |
| Intercept | 90.0659 | 6.0996 | 0.0000 |
| Age | -0.0486 | 0.4001 | 0.9032 |
| Sex | 2.6680 | 2.4359 | 0.2742 |
| cohort1994 | 9.8845 | 2.7542 | 0.0004 |
| cohort2004 | 4.6125 | 3.4957 | 0.1880 |
| Pancreatic insufficiency | -3.5169 | 6.0077 | 0.5587 |
| *P. aeruginosa* | -1.2209 | 0.2877 | 0.0000 |
| *S. maltophilia* | -0.7614 | 0.3307 | 0.0213 |
| *B. cepacia* | -1.5363 | 0.4713 | 0.0011 |
| *A. xylosoxidans* | -1.4752 | 0.3799 | 0.0001 |
| MABSC | -2.1383 | 0.6416 | 0.0009 |
| Cleared MABSC | 2.8336 | 1.2876 | 0.0278 |
| MAC | -0.6959 | 1.0464 | 0.5060 |
| Cleared MAC | 2.1513 | 1.6266 | 0.1860 |
| Age*sex | -0.1041 | 0.1793 | 0.5615 |
| Age*cohort1994 | -0.5269 | 0.2166 | 0.0150 |
| Age*cohort2004 | 1.0865 | 0.6789 | 0.1095 |
| Age*pancreatic insufficiency | -0.6061 | 0.4041 | 0.1337 |

**Table E3: Parameter estimates with standard errors and p-values for model with only data for patients who fulfill the ATS/IDSA criteria**

|  | Parameter estimate | Standard error | p-value |
| --- | --- | --- | --- |
| Intercept | 90.4966 | 6.0681 | 0.0000 |
| Age | -0.2841 | 0.3267 | 0.3846 |
| Sex | 2.2885 | 2.1954 | 0.2978 |
| cohort1974 | -12.4537 | 3.0127 | 0.0000 |
| cohort1994 | 10.0587 | 2.9177 | 0.0006 |
| cohort2004 | 4.9639 | 3.6625 | 0.1760 |
| Pancreatic insufficiency | -4.1773 | 5.9543 | 0.4833 |
| *P. aeruginosa* | -0.9428 | 0.1454 | 0.0000 |
| *S. maltophilia* | -0.6715 | 0.2777 | 0.0156 |
| *B. cepacia* | -1.9539 | 0.2843 | 0.0000 |
| *A. xylosoxidans* | -1.5212 | 0.3340 | 0.0000 |
| MABSC | -2.3458 | 0.5101 | 0.0000 |
| Cleared MABSC | 1.9044 | 0.7557 | 0.0117 |
| MAC | -0.1164 | 0.5263 | 0.8250 |
| Cleared MAC | 1.1582 | 1.1983 | 0.3338 |
| Age*sex | 0.0330 | 0.1319 | 0.8023 |
| Age*cohort1974 | 0.5815 | 0.1716 | 0.0007 |
| Age*cohort1994 | -0.5720 | 0.2318 | 0.0136 |
| Age*cohort2004 | 1.0667 | 0.6866 | 0.1203 |
| Age*pancreatic insufficiency | -0.4149 | 0.3214 | 0.1968 |

**Table E4: Comparing model fit when adding infection interaction terms – none of these improve model fit**

| **Model** | **AIC** |
| --- | --- |
| Baseline as per E1 | 378166.2 |
| Baseline + Pseudomonas*Maltophilia | 378167.2 |
| Baseline + Pseudomonas*Burkholderia | 378167.9 |
| Baseline + Maltophilia*MABSC | 378166.9 |
| Baseline + Pseudomonas*(MABSC+MABSCoffset) | 378169.2 |

**References**

1. Diggle P, Heagerty P, Liang K-Y, Zeger SL. Analysis of longitudinal data (second edition). Oxford: Oxford University Press; 2002.

2. Fitzmaurice GM, Laird, N.M. and Ware, J.H. . Applied longitudinal analysis. New Jersey: Wiley; 2004.

3. Matern B. Spatial variation. Meddelanden fran statens skogsforsknings institut, stockholm. Band 49, number 5. 1960.

4. McKay RT, LeMasters GK, Hilbert TJ, Levin LS, Rice CH, Borton EK, Lockey JE. A long term study of pulmonary function among us refractory ceramic fibre workers. *Occupational and environmental medicine* 2011;68:89.

5. Szczesniak RD, McPhail GL, Duan LL, Macaluso M, Amin RS, Clancy JP. A semiparametric approach to estimate rapid lung function decline in cystic fibrosis. *Annals of epidemiology* 2013;23:771-777.
